# Phase angle is related to physical function in high-risk Dutch older adults: implications for sarcopenia screening

**DOI:** 10.1016/j.tjfa.2025.100071

**Published:** 2025-09-03

**Authors:** Pol Grootswagers, Alice Ricco, Paul Hulshof, Lisette de Groot

**Affiliations:** Division of Human Nutrition and Health, Wageningen University, P.O. Box 17, 6700, AA, Wageningen, Netherlands

**Keywords:** Elderly, Phase angle, Sarcopenia, Frailty, Physical function, Muscle loss

## Abstract

**Introduction:**

Sarcopenia, a progressive age-related loss of skeletal muscle mass and function, poses significant health risks in older adults. Phase angle (PhA), derived from bioimpedance analysis, has been proposed as an indicator of muscle quality and physical functioning. This study investigates the association between PhA and physical function, and its potential utility in case-finding phase of sarcopenia assessment based on EWGSOP2 functional cut-offs.

**Methods:**

This cross-sectional observational study used baseline data from two clinical trials involving Dutch older adults (≥65 years, n=228) at risk of malnutrition or frailty. PhA was measured using multi-frequency bioimpedance vector analysis. Physical functioning was assessed through handgrip strength, knee extension strength, chair rise test, and gait speed (4m and 400-m/6-min walk tests). Associations were evaluated using linear mixed models adjusted for age, gender, height, and lean body mass. Receiver-operating characteristic (ROC) analyses identified PhA thresholds for low performance based on EWGSOP2 cut-offs.

**Results:**

PhA was significantly associated with all performance outcomes in crude models. After adjustment, each unit increase in PhA was associated with a 43.5 ± 8.4 N increase in knee extension strength (P < 0.0001), a 1.5 ± 0.4 s reduction in chair rise time (P = 0.0004), and a 0.14 ± 0.02 m/s increase in gait speed (P < 0.0001). Associations with handgrip strength became non-significant after full adjustment. A PhA threshold of 5.4° showed high sensitivity (0.96) for detecting low physical performance via the chair rise test. However, misclassification rates exceeded 25 %.

**Conclusions:**

PhA is associated with physical function, particularly lower-body performance measures, but without muscle mass assessment, it cannot support a complete diagnosis of sarcopenia. It may be valuable as a case-finding tool in older adults at risk.

**Abbreviations**
6MWT: 6‑Minute Walk Test
BIS: Bioelectrical Impedance Spectrometry
BIVA: Bio‑impedance Vector Analysis
BMI: Body Mass Index
DXA: Dual‑energy X‑ray Absorptiometry
EWGSOP2: European Working Group on Sarcopenia in Older Adults (updated guidelines)
FFM: Fat‑Free Mass
PhA: Phase Angle
R: Resistance
SARC‑F: (Strength, Ambulation, Rising from a chair, stair Climbing and history of Falling. Questionnaire to diagnose sarcopenia.)
SPPB: Short Physical Performance Battery
WT: Walking Test
Xc: Reactance
Xc parallel: Parallel Reactance
Z200/Z5 ratio: Ratio of impedance measured at 200 kHz to that measured at 5 kHz

## 1 Introduction

Sarcopenia, defined as a progressive, age-related skeletal muscle disorder, is prevalent in around 10 % of older adults [1]. The presence of sarcopenia increases the risk of functional decline, falls and all-cause mortality [2], and the condition should therefore be detected and treated in early stages [3].

In the updated guidelines for sarcopenia diagnosis, published by the European Working Group on Sarcopenia in Older Adults (EWGSOP2), muscle strength is considered the primary parameter for case finding. Probable sarcopenia is identified when an individual shows low muscle strength, defined as reduced hand grip strength (men <27 kg, women <16 kg) or slow five-times chair rise performance (>15 s). The strength assessment is followed by a muscle mass measurement, often performed by bio-impedance measurements or DXA, to confirm presence of sarcopenia. Once sarcopenia is confirmed, severity is determined based on physical performance, with slow gait speed (≤0.8 m/s) [3].

The bioimpedance analysis performed in the confirmation phase provides, depending on the device used, a phase angle value (PhA). Possibly, this phase angle measurement could play a role in estimating physical functioning. Mathematically, PhA is defined as the arc tangent of the ratio between reactance to resistance – values that reflect the capacity of cell membranes to shortly store charges and therefore considered to reflect muscle mass and cell membrane integrity [4]. Higher PhA values of around 8.5° are observed in elite athletes [5], whereas low PhA values of around 2.0° are reported in severely malnourished older adults [6]. However, a clear relationship between PhA and physical functioning is not confirmed yet. Some studies suggest that a relationship exists between PhA and physical functioning in older adults [[7], [8], [9]], but others are inconclusive [10].

Given the biological plausibility linking PhA to cellular integrity and muscle quality [4,[11], [12], [13]], we hypothesize that PhA is positively associated with multiple domains of physical functioning in older adults. Unlike previous studies that focused on limited performance measures or lacked comprehensive adjustments, the present study examines these associations using validated physical performance tests in a well-characterized cohort. In addition, this study explores the screening potential of PhA in comparison to the 2019 EWGSOP2 strength and performance thresholds [3], providing new insights into its clinical utility.

A tight relation between PhA and physical functioning could mean that the practically feasible PhA assessment could play a role in physical function assessment – and perhaps reducing the sarcopenia diagnosis battery to one bio-impedance measurement. However, first careful assessment of the exact relationship between PhA and physical functioning in multiple cohorts of older adults is needed, as well as estimating the diagnostic value of PhA in sarcopenia assessment. Therefore, in this study, we investigate (1) the relationship between PhA and handgrip strength, chair rise test and gait speed in Dutch older adults and (2) the potential of PhA as a screening tool based on the 2019 strength and physical function cut-offs set by the European Working Group on Sarcopenia in Older Adults.

To explore this relationship, we conducted a cross-sectional observational analysis using baseline data from two clinical trials in older adults at risk of sarcopenia.While this study evaluates strength and function, muscle mass was not assessed, and therefore no formal diagnosis of sarcopenia is made.

## 2 Methods

**Fig. 1:**
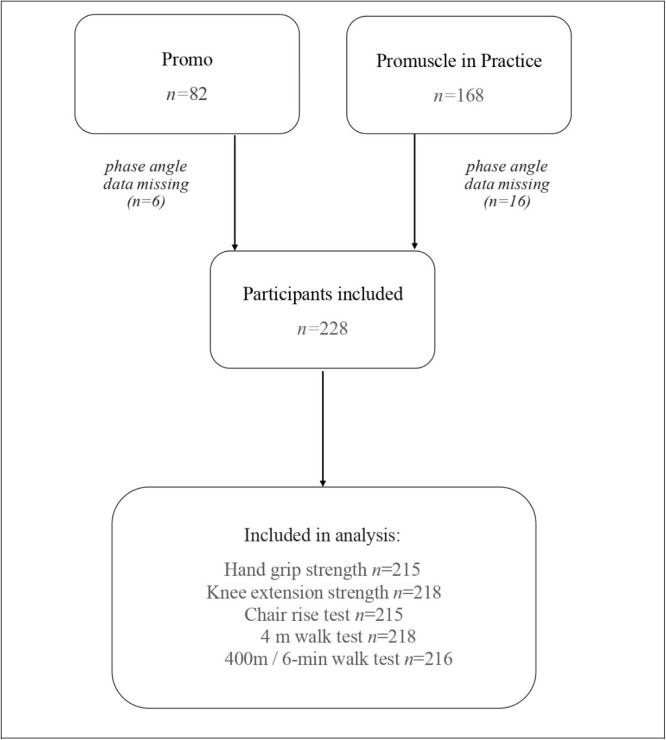
Flowchart of the participants.

We analyzed baseline data from two clinical trials (Promo: NCT02683720 and ProMuscle in Practice: NTR6038). Full inclusion criteria of these trials are described elsewhere [14,15]. In short, the Promo trial included 82 older adults (≥65 y) who were (at risk of being) malnourished, and excluded those involved in resistance exercise, using diabetes medication, with severely reduced kidney function and with a life-expectancy shorter than 12 months. The Promuscle in Practice trial included 168 older adults (≥65 y) who were (pre-) frail based on Fried’s criteria [16] or who experienced difficulties in daily activities in combination with physical inactivity, and excluded those with diabetes, severe heart failure, recent surgery, renal insufficiency, COPD and cancer. The flow of participants is shown in Fig. 1.

We focused on older adults at risk of malnutrition or frailty because these populations are especially vulnerable to declines in muscle quality and physical function, and at increased risk of developing sarcopenia.

This study used a cross-sectional design, analyzing baseline data from both trials to explore associations between phase angle and measures of physical functioning, and to assess the diagnostic accuracy of phase angle using EWGSOP2 criteria.

It is important to note that we did not include muscle mass measurements in the operational definition of sarcopenia in this study. Therefore, this analysis does not reflect a formal diagnosis but rather evaluates phase angle as a potential screening marker during the initial case-finding stage.

## 3 Phase angle

Phase angle and changes in intra- and extracellular water were measured by multi-frequency bioimpedance analysis (BIA, SFB7, Impedimed Limited, Pinkeba, QLD, Australia).

In both intervention trials, Bioelectrical Impedance Analysis (BIA) was conducted using the SFB7 device, with the Phase Angle (PhA) parameter recorded directly from the device at 50 kHz frequency. During measurements, participants lay down with their arms and legs positioned apart from their body, and they wore no metal or conductive items. Electrodes were placed following the standard tetrapolar setup [17]: one electrode was positioned between the wrist’s radial styloid and ulnar prominence, with a second electrode placed 5 cm lower, just below the middle finger’s knuckle. A third electrode was attached between the ankle’s lateral and medial malleolus, while the fourth electrode was placed 5 cm lower, directly below the index toe. Before electrode attachment, both the hand and foot were cleaned with alcohol, and any excessive hair was removed; electrodes were not applied over birthmarks or scars. BIA measurements were taken on the participant's right side. Participants were required to have a light breakfast before measurements, and in the ProMuscle study, participants were also instructed to urinate beforehand. Consequently, subject preparation was not uniform and variations in hydration status between the study groups are anticipated, potentially influencing BIA measurement outcomes [18,19].

## 4 Physical function

Isometric knee extension and flexion strength (measured in Newtons) were assessed using a handheld dynamometer (MicroFET2, HOGGAN Scientific LLC, Salt Lake City, UT, USA). Participants sat on an examination table with their knees bent at a 90-degree angle. For stability, the examiner sat against the wall, provided consistent verbal encouragement, and applied counterforce to the dynamometer. The dynamometer was positioned just above the ankle joint—on the front of the leg for extension and the back of the leg for flexion. Participants were instructed to gradually apply force within the first second after a standardized countdown ("3–2-1-GO") and then maintain maximum voluntary force for up to 4 s. Each participant performed a familiarization trial followed by six repetitions, alternating between legs. The peak force measurements from both the dominant and non-dominant legs were used for analysis, according to the manufacturer’s instructions.

Handgrip strength was measured with a hydraulic dynamometer (Jamar, Jackson, MI, USA), with results recorded to the nearest kilogram. Participants were seated in a chair without armrests, with their arms bent at a 90-degree angle. Each hand was tested three times in an alternating pattern, and the highest values for both dominant and non-dominant hands were analyzed, according to the manufacturer’s instructions.

Physical performance was evaluated using the Short Physical Performance Battery (SPPB), consisting of (1) a 4-meter walking test at a usual gait speed, (2) a repeated chair rise test, and (3) a balance test. Each test was scored from 0 to 4 according to the original SPPB protocol [20].

Lean body mass was assessed using dual-energy x-ray absorptiometry (DXA, Lunar Prodigy Advance; GE Health Care, Madison, WI, USA) in the same morning as BIA measurements were taken. Preparation and positioning of subjects were performed according to the manufacturer’s instructions.

Height was measured to the nearest 0.1 cm, at baseline, using a stadiometer.

## 5 Statistical analysis

Missing values for physical performance tests or phase angle were excluded listwise in the analyses. The number of valid observations for each outcome measure is provided in the corresponding tables (Table 2, Table 3, Table 4). Descriptive data are presented as mean ± SD or number ( %). Outcome data are presented as estimate ± standard error. Associations between phase angle and measures of physical functioning were assessed via three linear mixed models per outcome. To address potential confounding, three sequential models were used. The models adjusted for (Model 1) no covariates, (Model 2) age and gender, (Model 3) age, gender, height and lean body mass. Study-cohort was included as a random factor to model within cohort covariation. For handgrip strength and knee extension strength, the peak reading of all measurements was used, regardless of side. Walk tests were included as meters per second, and chair rise test as total seconds needed to complete five chair stands. Because of the assessment of five study outcomes, model assumptions were checked by visual inspection of residuals and Q-Q plots to assess normality and homoscedasticity, and the α level was set at 1 % (p < 0.01) to correct for multiple comparisons and reduce the risk of type I error. Graphics are made in GraphPad Prism 5 (GraphPad Software Inc., San Diego, CA, USA) and all analyses were performed in SAS, version 9.4 (SAS Institute, Inc., Cary, NC).

The secondary analysis of de-identified data was approved by the Medical Ethical Review Committee of Wageningen University under the ethical framework of the original studies.

## 6 Results

A total of 228 older adults were included, with a mean age of 75± 6 (Table 1). Between the two cohorts of participants, those in ProMO showed a significantly lower weight, BMI, fat free mass, fat percentage, and hand grip strength, but were faster on the longer walking test and the chair stand test.

**Table 1:** Baseline characteristics of the 228 independently-living older adults, enrolled in the ProMO (n=76) and ProMuscle (n=152) intervention trials.

	ProMO (n = 76)	ProMuscle (n = 152)	
Characteristics	Mean ± SD	Mean ± SD	p-value mean difference a
General			
Age (years)	74 ± 7	75 ± 6	0.527
Gender (n) ^b^			
Men	36	61	0.322
Women	40	91	
			
Anthropometry			
Weight (kg)	63.0 ± 10.8	76.4 ± 14.0	< 0.001
Height (cm)	168.9 ± 8.8	168.8 ± 9.3	0.921
BMI (kg/m^2^)	22.1 ± 3.2	26.8 ± 4.3	< 0.001
			
BIA			
PhA (°)	4.8 ± 0.7	5.0 ± 0.7	0.048
PhA/kg FFM (°/kg)	0.019 ± 0.022	0.018 ± 0.015	0.016
R (Ω)	563.3 ± 85.9	526.7 ± 74.7	0.001
Xc (Ω)	46.6 ± 8.0	45.5 ± 7.6	0.296
FFM (kg)	47 ± 9	52 ± 10	< 0.001
FFM-index (kg/m^2^)	16.4 ± 2.2	18.3 ± 2.5	< 0.001
Fat ( %)	25 ± 6	31 ± 7	< 0.001
Hydration status ( %)	74.3 ± 2.6	74.6 ± 2.4	0.386
DXA Scan Lean body mass (kg)	46.6 ± 8.9	47.9 ± 9.2	0.307
Physical function			
6MWT / 400-m WT (m/s) c	1.17 ± 0.24	1.04 ± 0.19	< 0.001
Knee extension (N)	336.7 ± 102.2	317.7 ± 104.1	0.193
Handgrip strength (kg) d	22.6 ± 9.8	29.1 ± 11.8	< 0.001
5x CRT (s) e	10.9 ± 4.1	13.3 ± 3.6	< 0.001
4-m WT (s)	4.27 ± 1.21	4.15 ± 0.98	0.458

BMI, body mass index (weight in kilograms divided by height in meters squared); PhA/kg FFM, phase angle normalized to fat-free mass; R, resistance (Ω); Xc, reactance (Ω); FFM, fat free mass; FFM-index, fat-free mass divided by height squared (kg/m²); DXA, dual-energy x-ray absorptiometry; WT, walking test (s); CRT, chair rise test; (M)WT, (minute) walking test (m/s)

a Student’s T-test to compare means and Chi-square to compare proportions. ^b^ Presented as proportion ( %).

c ProMO study: n=73. In total 225 participants.

d ProMuscle study: n=149. In total 225 participants.

e ProMO study: n=74; ProMuscle study: n=151. In total 225 participants.

PhA was significantly related to all domains of physical functioning in the crude models (Table 2). After adjustments for confounding and multiplicity, the association between PhA and hand grip strength attenuated to insignificance. Per unit increase in PhA, knee extension strength improved with 43. 5 ± 8.4 N (P<0.0001), chair rise test with 1.5 ± 0.4 s (P=0.0004) and gait speed, both on short and longer distance, with 0.14 ± 0.02 m/s (P<0.0001).

**Table 2:** Association between phase angle and five measures of physical functioning.

	Handgrip strength (*n*=215)		Knee extension strength (*n*=218)		Chair rise test (*n*= 215)		4 m gait speed (*n*=218)		400 m (*n*=64) or 6 min (*n*=152) gait speed	
	β Phase angle	P-value	β Phase angle	P-value	β Phase angle	P-value	β Phase angle	P-value	β Phase angle	P-value
Model 1	5.1 ± 1.0 kg	<0.0001	74.2 ± 8.4 N	<0.0001	-1.7 ± 0.3 s	<0.0001	0.14 ± 0.02 m/s	<0.0001	0.15 ± 0.02 m/s	<0.0001
Model 2	1.3 ± 1.0 kg	0.130	40.6 ± 7.6 N	<0.0001	-1.2 ± 0.4 s	0.001	0.10 ± 0.02 m/s	<0.0001	0.10 ± 0.02 m/s	<0.0001
Model 3	2.1 ± 0.9 kg	0.026	43.5 ± 8.4 N	<0.0001	-1.5 ± 0.4 s	0.0004	0.14 ± 0.02 m/s	<0.0001	0.14 ± 0.02 m/s	<0.0001

Model 1. Adjusted for study-cohort.

Model 2. Adjusted for study-cohort, age and gender

Model 3. Adjusted for study-cohort, age, gender, height and lean body mass.

N, Newton

Subgroup analysis by gender showed generally consistent associations between PhAand physical function measures in both males and females (Supplementary Table S1). In females, PhA remained significantly associated with all five outcomes in fully adjusted models. In males, associations remained significant for gait speed and knee extension strength, while associations with handgrip strength and chair rise time were no longer significant.

In Table 3, participants are stratified according to the EWGSOP2 cut-offs for sarcopenia, revealing that individuals classified with low physical performance exhibited significantly lower PhA values compared to those with normal performance. Specifically, the low handgrip strength and chair rise groups showed mean PhA differences of 0.3° (P = 0.012 and P = 0.003, respectively), while the low gait speed group had an even larger difference of 0.7° (P < 0.0001).

**Table 3:** Phase angle per group split on EWGSOP2 cut-offs for sarcopenia assessment.

Handgrip strength	Low (<16 or 27 kg a, *n*=58)		Normal (*n*=167)		Difference	
	Mean ± SE	Range	Mean ± SE	Range	Mean ± SE	P-value
Phase Angle	4.7 ± 0.1	3.0 – 6.9	5.0 ± 0.1	3.1 – 6.7	0.3 ± 0.1	0.012
Chair rise Test	Low (>15 s, *n*=45)		Normal (*n*=180)		Difference	
	Mean ± SE	Range	Mean ± SE	Range	Mean ± SE	P-value
Phase Angle	4.6 ± 0.1	3.0 – 5.9	5.0 ± 0.1	3.1 – 6.9	0.3 ± 0.1	0.003
Gait speed ^b^	Low (≤0.8 m/s, *n*=37)		Normal (*n*=191)		Difference	
	Mean ± SE	Range	Mean ± SE	Range	Mean ± SE	P-value
Phase Angle	4.3 ± 0.1	3.0 – 5.9	5.0 ± 0.0	3.5 – 6.9	0.7 ± 0.1	<0.0001

a The 16 kg cut-offs is for females, the 27 kg for males. ^b^ Based on the 4-meter walk test.

Receiver-operating characteristic analyses (Table 4) further demonstrated that the ability of PhA to discriminate between individuals with low and normal physical function, as defined by EWGSOP2 functional cut-offs. Notably, a phase angle threshold of 5.4° yielded a high sensitivity of 0.96 for the chair rise test, albeit with low specificity (0.29), whereas the gait speed analysis showed a more balanced performance (AUC = 0.74, sensitivity = 0.65, specificity = 0.72). These trends are visually represented in the Supplementary Figure S1, where scatterplots with color-coded diagnostic outcomes illustrate a clear association between lower PhA values and impaired physical performance.

**Table 4:** Receiver-Operating characteristics for phase angle to estimate low physical functioning, based on EWGSOP2 cut-offs.

	AUC	P-value AUC (difference from 0.50)	Phase angle threshold	Sensitivity	Specificity
Low handgrip strength	0.62	P=0.007	4.5	0.47	0.81
Low chair rise test	0.63	P=0.004	5.4	0.96	0.29
Low gait speed	0.74	P<0.0001	4.6	0.65	0.72

EWGSOP2, European Working Group on Sarcopenia in Older Patients revised version. Cut-offs are:

Handgrip strength, <27 kg (male), <16 kg (female; Chair rise test, >15 s; gait speed, <0.8 m/s

## 7 Discussion

This study investigated the relationship between phase angle (PhA) and physical performance tests in Dutch older adults at risk of malnutrition or frailty and explored the potential utility of PhA as a screening tool in the context of sarcopenia case-finding. Our results demonstrate that PhA is positively associated with several indicators of physical function, including knee extension strength, chair rise test, and gait speed. While an association with handgrip strength was observed in crude models, this relationship was attenuated and became non-significant after adjustment for covariates.

The present study reinforces the results of previous work showing an association between PhA and measures of physical performance [[21], [22], [23], [24]]. However, in contrast to some reports showing stronger associations [25], we observed that the relationship between PhA and handgrip strength was attenuated and no longer significant after full adjustment for covariates. Although hand grip tests are reliable for measuring strength, they may not accurately reflect overall physical function in the elderly, particularly those with conditions such as osteoarthritis that affect hand mobility and grip capacity [26]. Furthermore, handgrip strength has limited sensitivity in detecting changes in overall muscle strength over time [27]. Analyses divided by sex revealed that these associations were generally consistent between males and females, although slightly more robust in females. In females, PhA was significantly associated with all five physical function outcomes. In males, associations remained significant for gait speed and knee extension, while relationships with handgrip strength and chair rise time became non-significant. These differences may reflect sex-specific differences in body composition, fat distribution, or muscle quality.

Since our this study did not evaluate muscle mass assessment, it does not meet the full diagnostic criteria for sarcopenia as defined by EWGSOP2. As such, our conclusions are limited to the potential screening role of PhA for identifying individuals who may require further evaluation. To assess this screening potential, we evaluated the diagnostic value of PhA using ROC analysis. A PhA of 5.4° showed high sensitivity (0.96) in predicting low physical functioning based on the chair rise test, indicating its potential utility for ruling out impaired function in early screening [28]. However, the overall discriminative ability was only moderate, as reflected by AUC values ranging from 0.62 to 0.74. Additionally, all cut-offs tested produced misclassification rates greater than 25 %, which limits the clinical accuracy of PhA as a stand-alone diagnostic tool [29]. Therefore, although PhA may contribute to case-finding strategies, it should be interpreted cautiously and used in conjunction with other tools such as the SARC-F questionnaire to improve overall diagnostic performance, as recommended by the EWGSOP2 guidelines [3]. PhA measurements by bioelectrical impedance are rapid, noninvasive, and unnoticeable to subjects, and provide additional information on body composition as well [18]. The SARC-F questionnaire could optionally be used alongside PhA to improve specificity, as it has high diagnostic specificity but low sensitivity.

Another study has suggested a PhA cutoff for the diagnosis of sarcopenia ranges from 3.55° to 5.05°, however, sensitivity and specificity were moderate. A cutoff of 4.55° showed approximately 70 % sensitivity in geriatric patients, while a cutoff of 5.05° in cirrhotic patients showed 73.3 % sensitivity [11]. Although these thresholds are moderately effective, they fall short of the 96 % sensitivity observed in our study with a cutoff of 5.4° The higher threshold may be suitable for detecting early functional decline in older adults at risk, especially in clinical setting were higher sensitivity is preferred [11].

The reason why PhA may help identify individuals at risk of sarcopenia is that it reflects cellular integrity and muscle quality [[11], [12], [13]], both of which contribute to the declines in physical function in the elderly [30,31]. Our results support previous evidence showing that phase angle value can reflect physical function. Given this relationship, PhA could potentially be usefule in the early stages of sarcopenia case-finding. However, our results show that for any PhA cut-off, the proportion of miscategorized individuals always exceeded 25 %, underscoring its limited precision when used alone. Despite the high sensitivity associated with a value of 5.4°, our study highlights that the diagnostic classification of PhA remains challenging due to the high overall error rate and indicates that PhA should not be used as a stand-alone tool to determine sarcopenia risk. Instead, PhA may serve as a preliminary screening measure to flag individuals who would benefit from full diagnostic assessment, including strength and muscle mass measurements.

This study has limitations. The study sample includes elderly Dutch people who are frail or at risk of malnutrition. Although this group is clinically relevant for the study of sarcopenia, the findings may not be generalizable to healthier or more diverse populations. Additionally, ethnic and population-specific differences in body composition may limit the applicability of standard PhA cut-off points, making it difficult to apply the PhA for sarcopenia screening without context-specific validation. Another limitation is the potential variability in PhA measurements due to differences in bioimpedance measurement protocols between cohorts, including fasting status and urination instructions, which may have affected hydration level. Although study cohort was included as a random factor, residual measurement bias cannot be excluded. Moreover, PhA is inherently influenced by device type, electrode placement, and participant hydration, all of which may impact the consistency and replicability of BIA-derived values [4,17,32,33].

The 5.4° PhA threshold identified in this study was derived from a high-risk population and may not be generalizable to healthier or community-dwelling older adults. Diagnostic thresholds are likely to vary depending on health status, ethnicity, and body composition, and should therefore be validated in more diverse populations before broader clinical application. Since this study was cross-sectional, causal inferences cannot be made. Longitudinal research is required to determine whether changes in phase angle precede or follow declines in physical function associated with sarcopenia. Future investigations should assess the predictive value of PhA for monitoring physical decline over time and establish thresholds that signal critical functional deterioration, thereby guiding early interventions. Moreover, exploring the combined diagnostic utility of PhA and the SARC‑F questionnaire may improve case identification, balancing high sensitivity with improved specificity. Finally, additional bioimpedance parameters—such as lean mass and fat‑free mass—warrant further examination in relation to physical function and sarcopenia diagnosis.

In conclusion, our findings indicate that phase angle is moderately associated with key measures of lower-body physical functioning in high-risk older adults. Although a cutoff of 5.4° demonstrated high sensitivity in detecting low physical performance, the overall misclassification rate suggests that phase angle alone is not sufficient for accurate sarcopenia screening. Nevertheless, it may serve as a practical case-finding tool to identify individuals at risk who may benefit from further diagnostic assessment, particularly when combined with other diagnostic methods such as the SARC‑F questionnaire.

## CRediT authorship contribution statement

**Pol Grootswagers:** Writing – original draft, Validation, Supervision, Methodology, Investigation, Data curation, Conceptualization. **Alice Ricco:** Writing – original draft, Formal analysis. **Paul Hulshof:** Writing – review & editing, Supervision. **Lisette de Groot:** Writing – review & editing, Supervision.

## Declaration of competing interest

The authors declare that they have no known competing financial interests or personal relationships that could have appeared to influence the work reported in this paper.

## Appendix Supplementary materials

Image, application 1
