# Supplementary material for: Phase angle is related to physical function in high-risk Dutch older adults: implications for sarcopenia screening
Source: J Frailty Aging. 2025 Sep 3;14(5):100071. doi: 10.1016/j.tjfa.2025.100071 (PMC12446561; doi:10.1016/j.tjfa.2025.100071)
Supplement: Supplementary file 1 [file mmc1.docx]

**SUPPLEMENTARY MATERIAL**

| **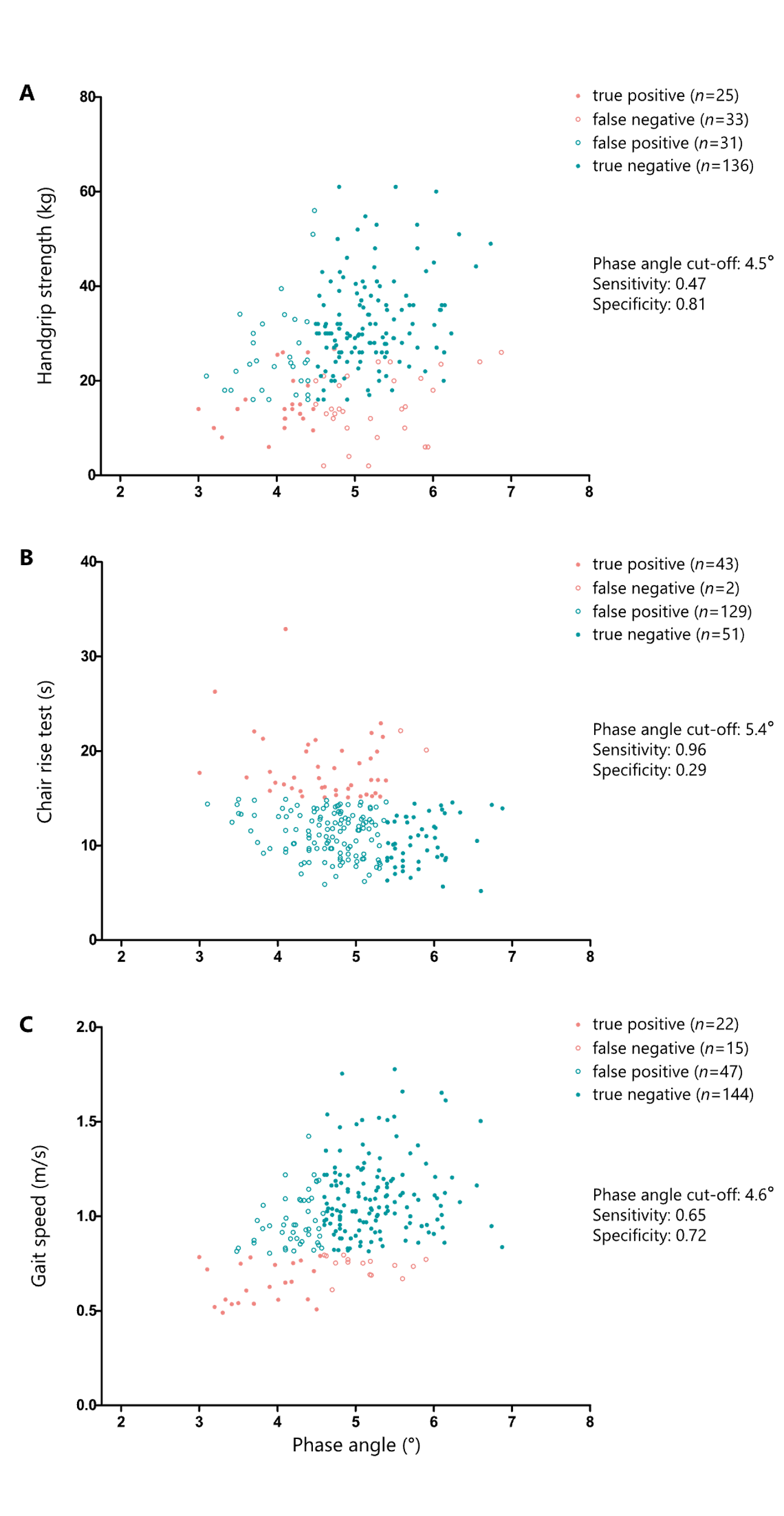** |
| --- |
| **Supplementary Figure S1.**  Associations between phase angle and measures of physical functioning, with observations colored based on diagnostic outcome. Phase angle cut-offs were based on Receiver-Operating characteristics and were 4.5 for the assessment of low-handgrip strength (**1A**), 5.4 for slow chair-rise test (**1B**), and 4.6 for slow gait speed (**1C**). |

| **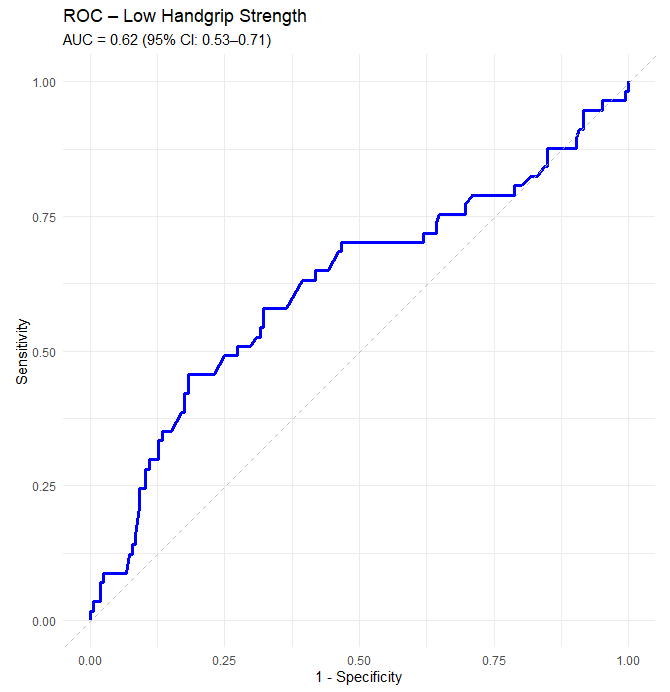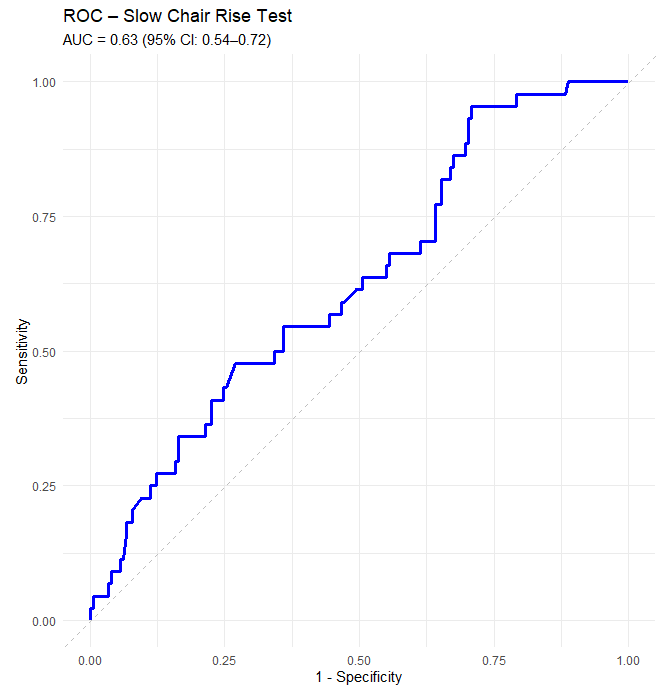**  **A**  **B**  **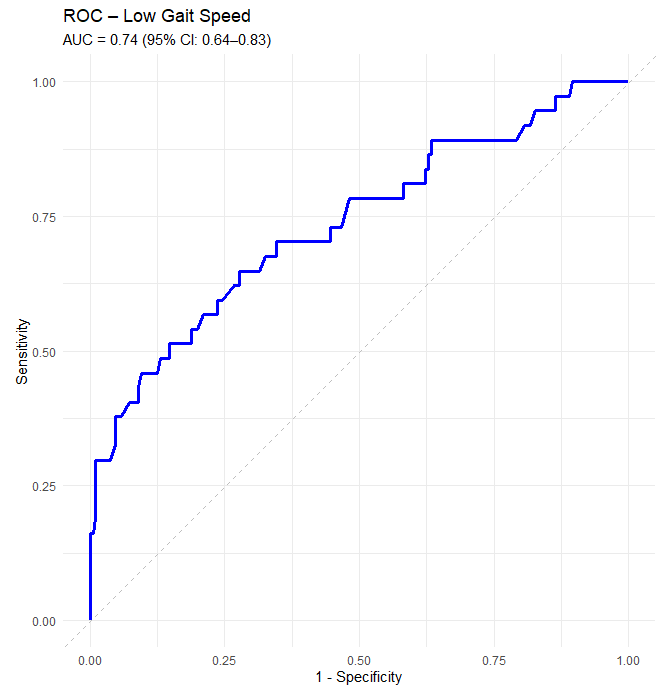**  **C** |
| --- |
| **Supplementary Figure S2.**  Receiver-Operating characteristics for phase angle to estimate low physical functioning, based on EWGSOP2 cut-offs. The Area Under the Curve was 0.62 for the assessment of low-handgrip strength (**2A**), 0.63 for slow chair-rise test (**2B**), and 0.74 for slow gait speed (**2C**). |

|  | **Supplementary Table S1.** Association between phase angle and five measures of physical functioning divided by sex. | | | | | | | | | | |
| --- | --- | --- | --- | --- | --- | --- | --- | --- | --- | --- | --- |
|  |  | Handgrip strength  (*n*=215) | | Knee extension strength (*n*=218) | | Chair rise test (*n*= 215) | | 4 m gait speed (*n*=218) | | 400 m (*n*=64) or  6 min (*n*=152) gait speed | |
| Gender |  | β Phase angle | P-value | β Phase angle | P-value | β Phase angle | P-value | β Phase angle | P-value | β Phase angle | P-value |
|  | Model 1 | 2.8 ± 1.0 kg | 0.007 | 46.5 ± 8.2 N | <.0001 | -1.5 ± 0.5 s | 0.005 | 0.14 ± 0.03 m/s | <.0001 | 0.15 ± 0.03 m/s | <.0001 |
| Females | Model 2 | 2.3 ± 1.1 kg | 0.037 | 38.4 ± 8.6 N | <.0001 | -1.2 ± 0.6 s | 0.033 | 0.13 ± 0.03 m/s | <.0001 | 0.12 ± 0.03 m/s | <.0001 |
|  | Model 3 | 2.5 ± 1.1 kg | 0.034 | 40.1 ± 9.4 N | <.0001 | -1.7 ± 0.6 s | 0.006 | 0.16 ± 0.03 m/s | <.0001 | 0.16 ± 0.03 m/s | <.0001 |
|  | Model 1 | 2.7 ± 1.3 kg | 0.042 | 64.4 ± 11.5 N | <.0001 | -1.3 ± 0.4 s | 0.002 | 0.11 ± 0.03 m/s | <.0001 | 0.13 ± 0.03 m/s | <.0001 |
| Males | Model 2 | -0.1 ± 1.5 kg | 0.946 | 46.3 ± 14.0 N | <.0001 | -1.3 ± 0.5 s | 0.021 | 0.06 ± 0.04 m/s | 0.110 | 0.09 ± 0.03 m/s | <.0001 |
|  | Model 3 | 2.4 ± 1.5 kg | 0.121 | 56.8 ± 15.2 N | <.0001 | -1.1 ± 0.6 s | 0.079 | 0.09 ± 0.04 m/s | 0.036 | 0.09 ± 0.04 m/s | <.0001 |
|  | Model 1. Adjusted for study-cohort. | | | | | | | | | | |
|  | Model 2. Adjusted for study-cohort and age | | | | | | | | | | |
|  | Model 3. Adjusted for study-cohort, age, height and lean body mass. | | | | | | | | | | |
|  | N, Newton | | | | | | | | | | |
